# Pyrimidin-2-amine–1-phenyl­cyclo­pentane-1-carb­oxy­lic acid (1/1)

**DOI:** 10.1107/S1600536811003667

**Published:** 2011-02-02

**Authors:** Guangwen He, Srinivasulu Aitipamula, Pui Shan Chow, Reginald B. H. Tan

**Affiliations:** a Institute of Chemical and Engineering Sciences, A*STAR (Agency for Science, Technology and Research), 1 Pesek Road, Jurong Island, Singapore 627833; b Department of Chemical & Biomolecular Engineering, National University of Singapore, 4 Engineering Drive 4, Singapore 117576

## Abstract

In the crystal structure of the title co-crystal, C_4_H_5_N_3_·C_12_H_14_O_2_, the components are linked by N—H⋯O and O—H⋯N hydrogen bonds. Self-assembly of these dimeric units results in a four-component supra­molecular unit featuring a homosynthon between two mol­ecules of the pyrimidin-2-amine involving two N—H⋯O hydrogen bonds, and two heterosynthons between each one mol­ecule of pyrimidin-2-amine and 1-phenyl­cyclo­pentane-1-carb­oxy­lic acid involving N—H⋯O and O—H⋯N hydrogen bonds.

## Related literature

For the structure of pyrimidin-2-amine, see: Scheinbeim & Schempp (1976 ▶) and for the structure of 1-phenyl­cyclo­pentane-1-carb­oxy­lic acid, see: Margulis (1975 ▶). For mol­ecular co-crystals of pyrimidin-2-amine, see: Serafin & Wheeler (2007 ▶); Shan *et al.* (2002 ▶); Goswami *et al.* (1999*a* ▶,*b* ▶, 2000 ▶); Chinnakali *et al.* (1999 ▶); Lynch *et al.* (1997 ▶). For a salt of 2-amino­pyridine and 1-phenyl-1-cyclo­propane­carb­oxy­lic acid, see: He *et al.* (2010 ▶). For a recent screening study for co-crystal and salt formation using pulse-gradient spin–echo nuclear magnetic resonance, see: He *et al.* (2009 ▶).
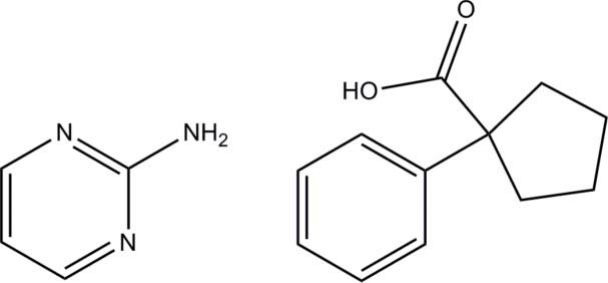


## Experimental

### 

#### Crystal data

C_4_H_5_N_3_·C_12_H_14_O_2_
*M* *_r_* = 285.34
Monoclinic, 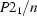

*a* = 9.1461 (18) Å
*b* = 10.490 (2) Å
*c* = 15.474 (3) Å
β = 98.14 (3)°
*V* = 1469.7 (5) Å^3^
*Z* = 4
Mo *K*α radiation
μ = 0.09 mm^−1^
*T* = 110 K
0.44 × 0.44 × 0.22 mm

#### Data collection

Rigaku Saturn 70 CCD area-detector diffractometer
Absorption correction: multi-scan (Blessing, 1995 ▶) *T*_min_ = 0.963, *T*_max_ = 0.981
20335 measured reflections
3641 independent reflections
3516 reflections with *I* > 2σ(*I*)
*R*_int_ = 0.036

#### Refinement

*R*[*F* ^2^ > 2σ(*F* ^2^)] = 0.068
*wR*(*F* ^2^) = 0.178
*S* = 1.21
3641 reflections
202 parameters
1 restraint
H atoms treated by a mixture of independent and constrained refinement
Δρ_max_ = 0.28 e Å^−3^
Δρ_min_ = −0.27 e Å^−3^

### 

Data collection: *CrystalClear* (Rigaku, 2007 ▶); cell refinement: *CrystalClear*; data reduction: *CrystalClear*; program(s) used to solve structure: *SHELXS97* (Sheldrick, 2008 ▶); program(s) used to refine structure: *SHELXL97* (Sheldrick, 2008 ▶); molecular graphics: *X-SEED* (Barbour, 2001 ▶); software used to prepare material for publication: *SHELXL97*.

## Supplementary Material

Crystal structure: contains datablocks I, global. DOI: 10.1107/S1600536811003667/ng5101sup1.cif

Structure factors: contains datablocks I. DOI: 10.1107/S1600536811003667/ng5101Isup2.hkl

Additional supplementary materials:  crystallographic information; 3D view; checkCIF report

## Figures and Tables

**Table 1:** Hydrogen-bond geometry (Å, °)

*D*—H⋯*A*	*D*—H	H⋯*A*	*D*⋯*A*	*D*—H⋯*A*
O2—H6⋯N1	0.87 (2)	1.79 (2)	2.653 (2)	173 (3)
N3—H5⋯O1	0.90 (3)	2.08 (3)	2.966 (2)	168 (2)
N3—H1⋯N2^i^	0.88 (3)	2.13 (3)	3.006 (2)	173 (2)

Symmetry code: (i) 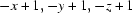.

**Fig. 1.**
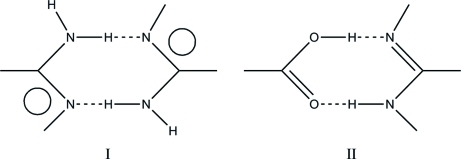
pyrimidin-2-amine–pyrimidin-2-amine supramolecular homosynthon (I) and pyrimidin-2-amine–carboxylic acid supramolecular heterosynthon (II).

**Fig. 2.**
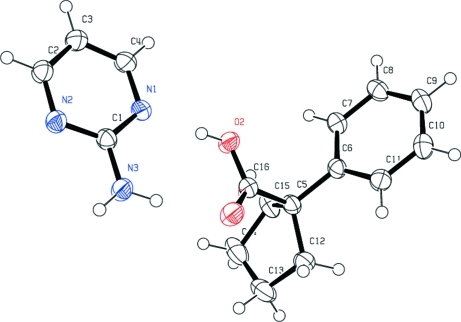
The molecular structures of pyrimidin-2-amine and 1-phenyl-1-cyclopropentanecarboxylic acid, with atom labels and 50% probability displacement ellipsoids for non-H atoms.

**Fig. 3.**
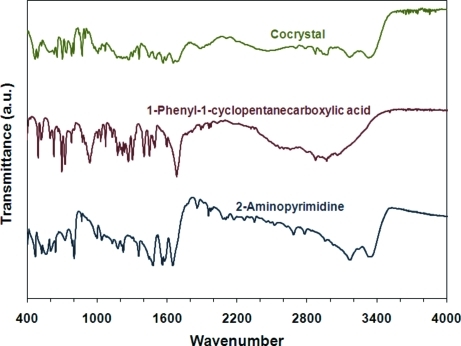
F T—IR spectra for pyrimidin-2-amine, 1-phenylcyclopentane-1-carboxylic acid and the 1/1 cocrystal of them, respectively.

**Fig. 4.**
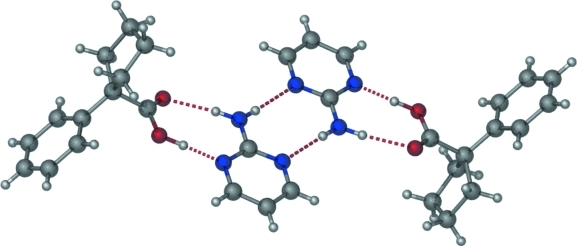
A four-component supramolecular unit that features N–H···O and O–H···N heterosynthon interactions, and O–H···O homosynthon interaction in the crystal structure of the title cocrystal.

**Fig. 5.**
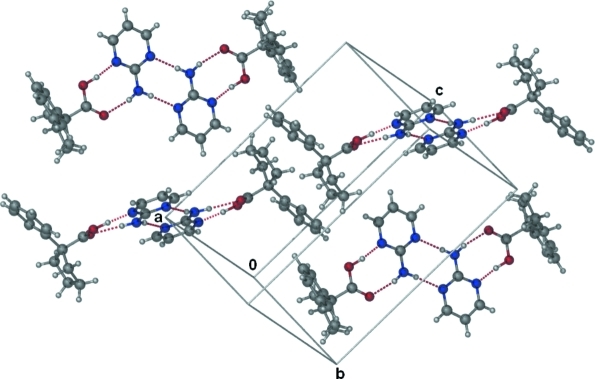
Part of the crystal structure of the title cocrystal, showing the arrangement of the four-component supramolecular units.

## supplementary crystallographic information

### Comment

An analysis of the crystal structure of pyrimidin-2-amine reveals that it forms a homosynthon (I) involving two N–H···N hydrogen bonds (Scheinbeim and Schempp, 1976). However, when it is cocrystallized with the molecules possessing at least one carboxylic acid group in the structure, it forms a pyrimidin-2-amine–carboxylic acid supramolecular heterosynthon (II) (Fig. 1) involving two hydrogen bonds, namely N–H···O and O–H···N. These strong hydrogen bonds are preferred over potential alternative arrangements and play a significant role in structure-directing (Shan *et al.*, 2002). We have chosen pyrimidin-2-amine and 1-phenylcyclopentane-1-carboxylic acid for cocrystallization experiment as an extension work to our previous study on screening for molecular cocrystals and salts (He *et al.*, 2009).

The crystal structure of the title cocrystal contains one molecule of pyrimidin-2-amine and one molecule of 1-phenylcyclopentane-1-carboxylic acid in the crystallographic asymmetric unit (Fig. 2). The identity of the cocrystal was confirmed by Fourier Transform Infrared (FT—IR) spectrum which showed carboxylic acid O—H stretching band at 3167 cm^-1^ and carbonyl stretching band at 1685 cm^-1^ (Fig. 3). Two pyrimidin-2-amine molecules that are related by an inversion center form the synthon I involving N–H···O (N···O = 3.006 (2) Å) hydrogen bonds. Two 1-phenylcyclopentane-1-carboxylic acid molecules hydrogen bond to either side of the dimeric motif involving synthon II which is sustained by N–H···O (N···O = 2.966 (2) Å) and O–H···O (O···O = 2.653 (2) Å) hydrogen bonds and forms a four-component supramolecular unit (Fig. 4). These four-component supramolecular units self assemble in the crystal structure *via* several weak C–H···O interactions (Fig. 5).

### Experimental

0.0957 g (1 mmol) of pyrimidin-2-amine (Alfa Aesar, 99%) and 0.1909 g (1 mmol) of 1-phenylcyclopentane-1-carboxylic acid (Alfa Aesar, 98%) and were dissolved into 7.6 ml of ethyl acetate (Fisher Scientific, HPLC). Solution was then filtered through a 0.22µm PTFE filter. Filtered solution was finally sealed with Parafilm and small holes were made to allow solvent to slowly evaporate. The block-shaped crystal (0.44 × 0.44 × 0.22 mm) suitable for single-crystal X-ray diffraction (Rigaku Saturn 70 CCD area detector with Mo *K*_α_ radiation = 0.71073 Å at 50 kV and 40 mA) was collected after one day. Fourier Transform Infrared (FT—IR) experiments were performed using Bio-Rad spectrometer (FTS3000MX) to confirm whether the resulting molecular complex is a cocrystal or a salt.

### Refinement

H atoms bonded to N and O atoms were located in a difference map and allowed to ride on their parent atoms in the refinement cycles.The O2—H6 bond distance which was found to be long in the normal refinement cycles was fixed using *DFIX* command in *SHELX*. Other H atoms were positioned geometrically and refined using a riding model.

### Crystal data

**Table:**

C_4_H_5_N_3_·C_12_H_14_O_2_	*F*(000) = 608
*M**_r_* = 285.34	*D*_x_ = 1.290 Mg m^−^^3^
Monoclinic, *P*2_1_/*n*	Mo *K*α radiation, λ = 0.71073 Å
*a* = 9.1461 (18) Å	Cell parameters from 4896 reflections
*b* = 10.490 (2) Å	θ = 1.9–31.1°
*c* = 15.474 (3) Å	µ = 0.09 mm^−^^1^
β = 98.14 (3)°	*T* = 110 K
*V* = 1469.7 (5) Å^3^	Block, colorless
*Z* = 4	0.44 × 0.44 × 0.22 mm

### Data collection

**Table:**

Rigaku Saturn 70 CCD area-detector diffractometer	3641 independent reflections
Radiation source: fine-focus sealed tube	3516 reflections with *I* > 2σ(*I*)
graphite	*R*_int_ = 0.036
ω scans	θ_max_ = 28.3°, θ_min_ = 2.4°
Absorption correction: multi-scan (Blessing, 1995)	*h* = −12→12
*T*_min_ = 0.963, *T*_max_ = 0.981	*k* = −13→13
20335 measured reflections	*l* = −20→20

### Refinement

**Table:**

Refinement on *F*^2^	Primary atom site location: structure-invariant direct methods
Least-squares matrix: full	Secondary atom site location: difference Fourier map
*R*[*F*^2^ > 2σ(*F*^2^)] = 0.068	Hydrogen site location: inferred from neighbouring sites
*wR*(*F*^2^) = 0.178	H atoms treated by a mixture of independent and constrained refinement
*S* = 1.21	*w* = 1/[σ^2^(*F*_o_^2^) + (0.0804*P*)^2^ + 0.5855*P*] where *P* = (*F*_o_^2^ + 2*F*_c_^2^)/3
3641 reflections	(Δ/σ)_max_ < 0.001
202 parameters	Δρ_max_ = 0.28 e Å^−^^3^
1 restraint	Δρ_min_ = −0.27 e Å^−^^3^

### Special details

**Table:**

Geometry. All e.s.d.'s (except the e.s.d. in the dihedral angle between two l.s. planes) are estimated using the full covariance matrix. The cell e.s.d.'s are taken into account individually in the estimation of e.s.d.'s in distances, angles and torsion angles; correlations between e.s.d.'s in cell parameters are only used when they are defined by crystal symmetry. An approximate (isotropic) treatment of cell e.s.d.'s is used for estimating e.s.d.'s involving l.s. planes.
Refinement. Refinement of *F*^2^ against ALL reflections. The weighted *R*-factor *wR* and goodness of fit *S* are based on *F*^2^, conventional *R*-factors *R* are based on *F*, with *F* set to zero for negative *F*^2^. The threshold expression of *F*^2^ > σ(*F*^2^) is used only for calculating *R*-factors(gt) *etc*. and is not relevant to the choice of reflections for refinement. *R*-factors based on *F*^2^ are statistically about twice as large as those based on *F*, and *R*- factors based on ALL data will be even larger.

### Fractional atomic coordinates and isotropic or equivalent isotropic displacement parameters (Å^2^)

**Table:**

	*x*	*y*	*z*	*U*_iso_*/*U*_eq_	
O2	0.04632 (14)	0.55094 (13)	0.70052 (8)	0.0344 (3)	
O1	0.22224 (15)	0.40633 (14)	0.73866 (9)	0.0397 (3)	
C16	0.12059 (18)	0.47154 (16)	0.75641 (11)	0.0281 (3)	
C6	−0.09084 (18)	0.42117 (16)	0.83708 (10)	0.0273 (3)	
C5	0.06701 (18)	0.47220 (17)	0.84621 (11)	0.0289 (4)	
C8	−0.35533 (19)	0.45531 (18)	0.80273 (12)	0.0337 (4)	
H8	−0.4356	0.5110	0.7842	0.040*	
C12	0.17880 (19)	0.3999 (2)	0.91211 (12)	0.0396 (4)	
H12A	0.2180	0.3247	0.8843	0.047*	
H12B	0.1319	0.3709	0.9625	0.047*	
C7	−0.21065 (18)	0.50062 (17)	0.81043 (11)	0.0305 (4)	
H7	−0.1936	0.5873	0.7972	0.037*	
C10	−0.2643 (2)	0.24866 (18)	0.84809 (12)	0.0362 (4)	
H10	−0.2821	0.1620	0.8610	0.043*	
C11	−0.1195 (2)	0.29396 (17)	0.85543 (12)	0.0332 (4)	
H11	−0.0395	0.2376	0.8731	0.040*	
C15	0.0807 (2)	0.6105 (2)	0.88196 (13)	0.0380 (4)	
H15A	0.0410	0.6727	0.8365	0.046*	
H15B	0.0279	0.6204	0.9332	0.046*	
C9	−0.3820 (2)	0.32934 (19)	0.82208 (12)	0.0354 (4)	
H9	−0.4805	0.2985	0.8175	0.042*	
C13	0.3039 (2)	0.4964 (3)	0.94177 (14)	0.0523 (6)	
H13A	0.3269	0.4975	1.0062	0.063*	
H13B	0.3945	0.4731	0.9172	0.063*	
C14	0.2473 (2)	0.6273 (2)	0.90771 (16)	0.0517 (6)	
H14A	0.2684	0.6930	0.9537	0.062*	
H14B	0.2944	0.6530	0.8566	0.062*	
H6	0.082 (3)	0.557 (3)	0.6516 (13)	0.066 (8)*	
C2	0.24306 (19)	0.66091 (17)	0.39920 (12)	0.0318 (4)	
H2	0.2760	0.6860	0.3462	0.038*	
N3	0.36606 (18)	0.47957 (17)	0.58582 (11)	0.0391 (4)	
N1	0.14713 (15)	0.58831 (14)	0.54997 (9)	0.0299 (3)	
N2	0.33123 (16)	0.58740 (14)	0.45437 (10)	0.0314 (3)	
C3	0.1056 (2)	0.70251 (18)	0.41554 (12)	0.0345 (4)	
H3	0.0445	0.7554	0.3757	0.041*	
C4	0.06304 (19)	0.66250 (18)	0.49318 (12)	0.0346 (4)	
H4	−0.0304	0.6890	0.5068	0.041*	
H5	0.331 (3)	0.447 (2)	0.6325 (17)	0.051 (7)*	
C1	0.27973 (18)	0.55295 (16)	0.52886 (11)	0.0285 (3)	
H1	0.451 (3)	0.454 (2)	0.5716 (16)	0.051 (7)*	

### Atomic displacement parameters (Å^2^)

**Table:**

	*U*^11^	*U*^22^	*U*^33^	*U*^12^	*U*^13^	*U*^23^
O2	0.0317 (6)	0.0434 (7)	0.0300 (6)	0.0106 (5)	0.0113 (5)	0.0085 (5)
O1	0.0385 (7)	0.0472 (8)	0.0361 (7)	0.0150 (6)	0.0146 (6)	0.0093 (6)
C16	0.0247 (7)	0.0303 (8)	0.0297 (8)	−0.0005 (6)	0.0056 (6)	0.0009 (6)
C6	0.0259 (7)	0.0322 (8)	0.0249 (8)	−0.0013 (6)	0.0071 (6)	−0.0011 (6)
C5	0.0226 (7)	0.0380 (9)	0.0268 (8)	−0.0023 (6)	0.0055 (6)	−0.0003 (6)
C8	0.0249 (8)	0.0410 (9)	0.0361 (9)	0.0003 (7)	0.0070 (7)	−0.0050 (7)
C12	0.0264 (8)	0.0626 (13)	0.0297 (9)	0.0001 (8)	0.0039 (7)	0.0093 (8)
C7	0.0279 (8)	0.0307 (8)	0.0336 (9)	−0.0016 (6)	0.0074 (6)	−0.0031 (7)
C10	0.0408 (10)	0.0329 (9)	0.0364 (9)	−0.0097 (7)	0.0102 (7)	−0.0021 (7)
C11	0.0331 (9)	0.0333 (9)	0.0336 (9)	0.0005 (7)	0.0058 (7)	0.0018 (7)
C15	0.0305 (9)	0.0449 (10)	0.0402 (10)	−0.0110 (7)	0.0106 (7)	−0.0113 (8)
C9	0.0303 (8)	0.0436 (10)	0.0339 (9)	−0.0108 (7)	0.0104 (7)	−0.0084 (7)
C13	0.0274 (9)	0.0954 (18)	0.0335 (10)	−0.0092 (10)	0.0018 (8)	−0.0042 (11)
C14	0.0357 (10)	0.0700 (15)	0.0500 (12)	−0.0211 (10)	0.0082 (9)	−0.0156 (11)
C2	0.0328 (8)	0.0344 (9)	0.0292 (8)	0.0018 (7)	0.0077 (6)	0.0018 (7)
N3	0.0306 (8)	0.0513 (10)	0.0383 (9)	0.0149 (7)	0.0150 (7)	0.0168 (7)
N1	0.0238 (6)	0.0371 (8)	0.0294 (7)	0.0027 (5)	0.0065 (5)	0.0024 (6)
N2	0.0294 (7)	0.0340 (7)	0.0320 (8)	0.0035 (6)	0.0087 (6)	0.0040 (6)
C3	0.0304 (8)	0.0403 (10)	0.0326 (9)	0.0052 (7)	0.0045 (7)	0.0057 (7)
C4	0.0257 (8)	0.0437 (10)	0.0348 (9)	0.0070 (7)	0.0060 (6)	0.0041 (7)
C1	0.0256 (8)	0.0291 (8)	0.0316 (8)	0.0016 (6)	0.0066 (6)	0.0010 (6)

### Geometric parameters (Å, °)

**Table:**

O2—C16	1.318 (2)	C15—H15A	0.9900
O2—H6	0.869 (17)	C15—H15B	0.9900
O1—C16	1.217 (2)	C9—H9	0.9500
C16—C5	1.537 (2)	C13—C14	1.535 (4)
C6—C7	1.392 (2)	C13—H13A	0.9900
C6—C11	1.397 (2)	C13—H13B	0.9900
C6—C5	1.527 (2)	C14—H14A	0.9900
C5—C12	1.538 (2)	C14—H14B	0.9900
C5—C15	1.551 (3)	C2—N2	1.334 (2)
C8—C9	1.384 (3)	C2—C3	1.387 (2)
C8—C7	1.395 (2)	C2—H2	0.9500
C8—H8	0.9500	N3—C1	1.340 (2)
C12—C13	1.548 (3)	N3—H5	0.90 (3)
C12—H12A	0.9900	N3—H1	0.88 (3)
C12—H12B	0.9900	N1—C4	1.334 (2)
C7—H7	0.9500	N1—C1	1.352 (2)
C10—C9	1.384 (3)	N2—C1	1.355 (2)
C10—C11	1.397 (2)	C3—C4	1.380 (3)
C10—H10	0.9500	C3—H3	0.9500
C11—H11	0.9500	C4—H4	0.9500
C15—C14	1.530 (3)		
			
C16—O2—H6	113.4 (19)	C5—C15—H15B	111.1
O1—C16—O2	123.13 (16)	H15A—C15—H15B	109.1
O1—C16—C5	123.93 (16)	C10—C9—C8	119.54 (16)
O2—C16—C5	112.93 (14)	C10—C9—H9	120.2
C7—C6—C11	118.03 (15)	C8—C9—H9	120.2
C7—C6—C5	120.77 (15)	C14—C13—C12	106.50 (16)
C11—C6—C5	121.20 (15)	C14—C13—H13A	110.4
C6—C5—C16	109.44 (13)	C12—C13—H13A	110.4
C6—C5—C12	114.81 (15)	C14—C13—H13B	110.4
C16—C5—C12	109.27 (14)	C12—C13—H13B	110.4
C6—C5—C15	112.84 (14)	H13A—C13—H13B	108.6
C16—C5—C15	107.86 (14)	C15—C14—C13	105.13 (18)
C12—C5—C15	102.24 (15)	C15—C14—H14A	110.7
C9—C8—C7	120.06 (17)	C13—C14—H14A	110.7
C9—C8—H8	120.0	C15—C14—H14B	110.7
C7—C8—H8	120.0	C13—C14—H14B	110.7
C5—C12—C13	105.58 (17)	H14A—C14—H14B	108.8
C5—C12—H12A	110.6	N2—C2—C3	123.13 (16)
C13—C12—H12A	110.6	N2—C2—H2	118.4
C5—C12—H12B	110.6	C3—C2—H2	118.4
C13—C12—H12B	110.6	C1—N3—H5	120.2 (16)
H12A—C12—H12B	108.8	C1—N3—H1	118.2 (16)
C6—C7—C8	121.24 (16)	H5—N3—H1	121 (2)
C6—C7—H7	119.4	C4—N1—C1	117.03 (15)
C8—C7—H7	119.4	C2—N2—C1	116.58 (15)
C9—C10—C11	120.36 (17)	C4—C3—C2	115.97 (16)
C9—C10—H10	119.8	C4—C3—H3	122.0
C11—C10—H10	119.8	C2—C3—H3	122.0
C10—C11—C6	120.76 (17)	N1—C4—C3	122.94 (16)
C10—C11—H11	119.6	N1—C4—H4	118.5
C6—C11—H11	119.6	C3—C4—H4	118.5
C14—C15—C5	103.19 (17)	N3—C1—N1	117.64 (16)
C14—C15—H15A	111.1	N3—C1—N2	118.00 (15)
C5—C15—H15A	111.1	N1—C1—N2	124.36 (16)
C14—C15—H15B	111.1		

### Hydrogen-bond geometry (Å, °)

**Table:**

*D*—H···*A*	*D*—H	H···*A*	*D*···*A*	*D*—H···*A*
O2—H6···N1	0.87 (2)	1.79 (2)	2.653 (2)	173 (3)
N3—H5···O1	0.90 (3)	2.08 (3)	2.966 (2)	168 (2)
N3—H1···N2^i^	0.88 (3)	2.13 (3)	3.006 (2)	173 (2)

Symmetry codes: (i) −*x*+1, −*y*+1, −*z*+1.
